# Clinical trial assessing the safety of edoxaban with concomitant chemotherapy in patients with gynecological cancer-associated thrombosis (EGCAT study)

**DOI:** 10.1186/s12959-023-00500-8

**Published:** 2023-05-15

**Authors:** Tadashi Oride, Kenjiro Sawada, Aasa Shimizu, Yasuto Kinose, Tsuyoshi Takiuchi, Michiko Kodama, Kae Hashimoto, Eiji Kobayashi, Eiji Nakatani, Tadashi Kimura

**Affiliations:** 1 Department of Obstetrics and Gynecology, Osaka University Graduate School of Medicine, 2-15, Yamada-Oka, Suita City, Osaka, Japan; 2 Graduate School of Public Health, Shizuoka Graduate University of Public Health, Shizuoka, Japan

**Keywords:** Cancer associated thrombosis, Edoxaban, DOAC, Gynecological cancer, Trough concentration

## Abstract

**Background:**

Gynecological cancer is one of the highest risk factors for cancer-associated thrombosis (CAT). Although low-molecular-weight heparin (LMWH) is recommended as an anticoagulant for treating CAT, recent studies have shown that direct oral anticoagulants (DOACs) are an acceptable alternative. Patients with cancer require a series of chemotherapies concomitantly with DOAC administration; however, the extent to which these drugs influence DOAC blood concentrations is unknown. In this study, we measured the plasma concentration of edoxaban during chemotherapy for gynecological cancers to determine its safety.

**Methods:**

Patients histologically diagnosed with ovarian or uterine corpus cancer and CAT were recruited after primary surgery and before the initiation of postoperative adjuvant chemotherapy, including paclitaxel. Patients were administered edoxaban (30 or 60 mg) orally for CAT. The plasma concentrations of edoxaban and active factor Xa were determined and their percentage change before and after chemotherapy was calculated. Additionally, blood coagulation tests were analyzed.

**Results:**

Sixteen patients with gynecological cancer (12 with ovarian cancer and 4 with uterine corpus cancer) were enrolled. Among these, 15 samples were collected one day after chemotherapy initiation. During chemotherapy, the trough concentration of edoxaban changed from 17.6 ± 10.6 to 20.0 ± 15.6 ng/ml, and the mean percentage change in edoxaban concentration was 14.5%. Therefore, the trough concentrations of edoxaban, which represent excretion capacity, were not significantly increased by chemotherapy with paclitaxel. The area under the plasma edoxaban concentration–time curve and the active factor Xa concentration were also unaffected.

**Conclusion:**

Patients with CAT and ovarian or uterine corpus cancer administered edoxaban orally showed no significant increase in the trough concentration of edoxaban while undergoing chemotherapy. This suggests the safety of edoxaban use during the treatment of gynecological cancers.

**Trial registration:**

EGCAT study; Japan Registry of Clinical Trials, jRCTs051190024.

## Background

Patients with cancer are at an increased risk of thromboembolic disease because of their hypercoagulable state [1]. Its incidence in patients with cancer is four to seven times higher than those without cancer [2], and it is reported that 5–20% of all patients with cancer exhibit thromboembolisms [3]. In a recent Danish population-based cohort study, twelve-month incidence in the cancer cohort increased from 1.0% (95% CI (confidence interval): 0.9–1.2%) in 1997 to 3.4% (95% CI: 2.9–4.0%) in 2017, which was paralleled by the improved survival of patients and the increased use of computed tomography scans, chemotherapy, and targeted therapies [4].

Gynecological cancer has been identified by the Khorana Score as one of the highest risk factors for cancer-associated thrombosis (CAT) [5]. Three to ten percent of patients present with CAT at diagnosis, and its incidence increases up to 36% during cancer treatment, including the debulking surgeries and repeated chemotherapies [6, 7]. The risk factors for developing CAT are not only the presence of large pelvic masses which compress iliac veins but as also include comorbidities, immobilization, chemotherapy, targeted therapy (e.g., bevacizumab), surgeries including lymphadenectomy, and intravenous catheter; all these factors can contribute to the prothrombotic or hypercoagulable state, as defined by Virchow’s triad: stasis, hypercoagulability, and endothelial injury [7].

Low-molecular-weight heparin (LMWH), unfractionated heparin (UFH), and vitamin K antagonists (VKA) have been used to treat CAT. Previous phase III studies of patients with CAT have shown that the rate of recurrent thrombosis was lower with a six-month course of LMWH than with VKA, while the risk of bleeding was similar with both treatments [8, 9]. Therefore, LMWH has been recommended as an anticoagulant for the treatment of CAT in all major guidelines, including those of the American Society of Clinical Oncology, National Comprehensive Cancer Network, and American Thoracic Society [10]. However, the efficacy of LMWH beyond six months remains unclear, and LMWH therapy is burdensome, as it requires daily subcutaneous injections [11].

In 2010, direct oral anticoagulants (DOACs) such as apixaban, rivaroxaban, and edoxaban emerged. A randomized Phase III trial comparing each DOAC with VKA for the treatment of venous thromboembolism (VTE) showed that DOACs are not inferior to VKA in terms of treatment efficacy and recurrence rate; VTE treatment has shifted from using VKAs to DOACs owing to their efficacy, standardized dosing, reduced monitoring, less frequent follow-up, and fewer interactions with food or drugs [12]. More recently, clinical data supporting the use of DOACs in patients with cancer have become available. The comparison of an oral Factor Xa inhibitor with low molecular weight heparin in patients with cancer with venous thromboembolism (SELECT-D) study for rivaroxaban, the edoxaban for the treatment of cancer-associated venous thromboembolism (Hokusai VTE Cancer) study for edoxaban, and the apixaban for the treatment of venous thromboembolism associated with cancer (CARAVAGGIO) study for apixaban compared the efficacy of DOACs with that of LMWH, the first therapeutic choice for CAT treatment [11, 13, 14].

In the Hokusai-VTE Cancer study, edoxaban was a non-inferior treatment compared to dalteparin for recurrent VTE with major bleeding (12.8% vs. 13.5%, respectively) [11]. However, during six months of the study period, a significantly higher rate of major bleeding (6.9% vs. 4.0%) was observed with both treatments. Major bleeding episodes were mainly caused by upper gastrointestinal bleeding in patients with gastrointestinal cancer. In the SELECT-D study, major bleeding cumulative incidence at six months was 6% in the rivaroxaban group and 4% in the dalteparin group (HR, 1.83; 95% CI: 0.68–4.96) [13]. Although DOACs appear to be acceptable alternatives to LMWH for the treatment of CAT, several factors need to be considered to tailor anticoagulation management strategies for patients with active cancer [15]. Drug-drug interactions are important factors to be considered as systemic cancer-related therapies may interfere with DOACs. Potent inhibitors or inducers of P-glycoprotein and cytochrome p4503A4 (CYP3A4) are known to influence the metabolism of DOACs and potentially alter their efficacy and/or safety profiles [16]. However, the extent to which these drugs influence the blood concentrations of DOACs is unknown. Platinum and taxane agents are generally used for the initial treatment of gynecological cancers. Taxane agents such as paclitaxel are metabolized in the liver by CYP3A4 [17]. Therefore, the concomitant administration of DOAC and paclitaxel may increase the risk of major bleeding by affecting the blood concentration of DOAC. However, currently there are no studies which have reported the effects of chemotherapeutic agents on the metabolism of DOACs during chemotherapy. CAT is the second leading cause of death in patients with cancer receiving outpatient chemotherapy [3] and its incidence is high in gynecological cancer cases; therefore, the establishment of treatment strategies for CAT in gynecological cancer cases is clinically important.

In this study, we explore the safety of orally administrating edoxaban in patients with gynecological cancers undergoing chemotherapy, including paclitaxel, by evaluating the percentage change in the plasma concentration of edoxaban during treatment.

## Methods

### Participants

Participants included patients who were ≥ 20 and < 85 years-of-age, histologically diagnosed with ovarian or advanced uterine corpus cancer, and had VTE or pulmonary embolism (PE) which was confirmed by hematological tests (D-dimer) and imaging studies (lower-limb venous ultrasound and/or contrast-enhanced computed tomography). Patients with cervical and vulvar cancers were excluded from this study because of the different treatment strategies used, such as radiotherapy and concurrent chemoradiotherapy. After the primary surgeries were performed and the presence of malignancy was histologically confirmed, written informed consent was obtained before the initial postoperative adjuvant chemotherapy. As remaining tumors may affect the hypercoagulability of patients, those who underwent suboptimal surgeries were excluded. Patients who were expected to survive for at least six months from the time of consent were included in the study. Patients with a performance status > 2, impaired renal function (creatinine clearance less than 30 mL/min), abnormal liver function, weighing < 40 kg, with active bleeding or a high risk of bleeding, uncontrolled hypertension, history of hypersensitivity to DOACs, pregnancy or lactation, use of antiplatelet agents, history of venous thrombosis, complications of acute bacterial endocarditis, or history or treatment of atrial fibrillation were excluded from the study.

### Trial design and interventions

The clinical trial assessing the safety of edoxaban with concomitant chemotherapy in patients with gynecological cancer-associated thrombosis (EGCAT study) was a single-arm, comparative, open-label, uncontrolled treatment intervention study. This was a specified clinical trial under the Clinical Research Act in Japan and was sponsored by the Daiichi Sankyo Co., Ltd., (Tokyo, Japan). This study was conducted in accordance with the principles of the Declaration of Helsinki and the local regulations. The study protocol was reviewed and approved by the Osaka University Clinical Research Review Committee (CRB5180007). A summary of this clinical study was registered and published as jRCTs051190024 on 2019/06/03 in Janpan Registry of Clinical Trials (https://jrct.niph.go.jp/en-latest-detail/jRCTs051190024).

After obtaining written informed consent followed the primary surgeries, patients were orally administered an edoxaban (30 or 60 mg) (Daiichi Sankyo Co., Ltd., Tokyo, Japan) tablet daily after breakfast based on their weight and renal function. The edoxaban dosage was reduced to 30 mg in patients who met any of the following criteria: moderate renal impairment (creatinine clearance, 30–50 mL/min), body weight ≤ 60 kg, or concomitant use of potent P-glycoprotein inhibitors (such as erythromycin, cyclosporine, dronedarone, quinidine, or ketoconazole). Patients with ovarian cancer were scheduled to receive six cycles of carboplatin (area under the curve (AUC) = 5) with paclitaxel (175 mg/m^2^) with or without bevacizumab (15 mg/kg), while those with　uterine corpus cancer were scheduled to receive six cycles of carboplatin (AUC = 4), paclitaxel (150 mg/m^2^), and epirubicin (50 mg/m^2^) [18]. A pharmacokinetic study of edoxaban revealed that a steady-state blood concentration can be achieved after three days of treatment [19]; therefore, the scheduled postoperative chemotherapies were initiated more than three days after the administration of edoxaban. Blood samples were collected six times during chemotherapy. During the chemotherapy, all patients admitted the hospital. Edoxaban adherence was strictly checked by the pharmacologist in the ward during the admission. During the outpatient follow-up, participants presented self-reports regarding the adherence of their medication at every visit, and edoxaban adherence was strictly checked by attending physicians.

### Blood sampling

**Fig. 1:**
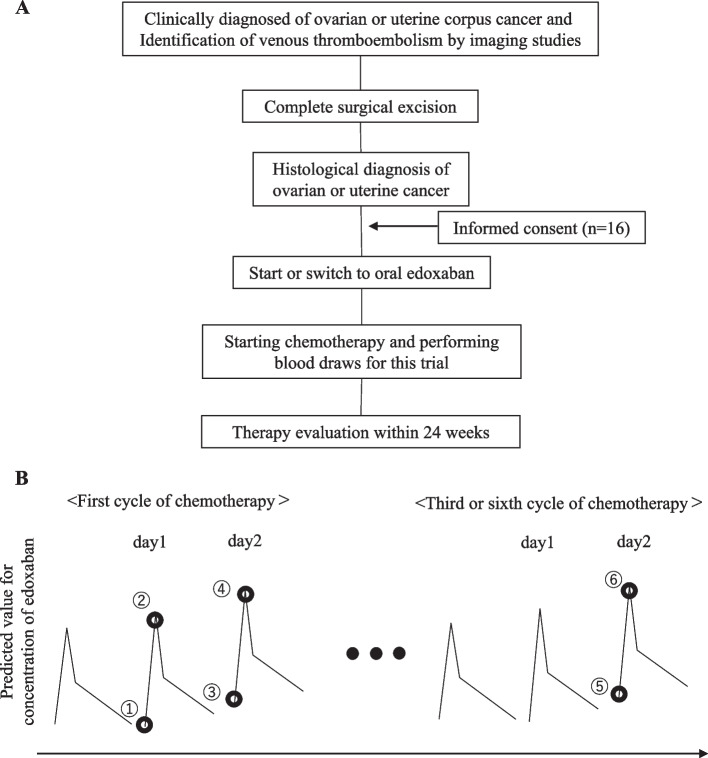
The overall schema of the trial and the schedule of blood sampling. **A** Trial design and intervention of this study. **B** The blood sampling schedule. Time point 1: Ctrough day1 (first day of chemotherapy before administrating edoxaban), time point 2: C_max_ day1 (first day of chemotherapy two hours after administrating edoxaban), time point 3: Ctrough day2 (day after chemotherapy initiation before administrating edoxaban), time point 4: C_max_ day2 (day after chemotherapy initiation two hours after administrating edoxaban), time point 5: Ctrough day2* (day after the final chemotherapy session before administrating edoxaban), time point 6: C_max_ day2* (day after the final chemotherapy session two hours after administrating edoxaban)

The overall schematic of the blood sampling schedule is shown in Fig. 1. The trough concentration (Ctrough) is defined as the concentration of a drug immediately before the next dose is administered and shows how a drug accumulates over time, providing valuable information about drug disposition [20]. On the first day of chemotherapy, blood was collected from patients after breakfast and before edoxaban administration (time point 1; Ctrough day1). Single oral doses of edoxaban result in peak plasma concentrations within 1.0–2.0 h of administration [19]; therefore, additional blood sampling was performed 2 h after the administration of edoxaban under physician supervision and was defined as maximum drug concentration (C_max_) day1 (time point 2). Thereafter, chemotherapy was initiated as described above. The third and fourth sets of blood sampling were performed the following day and defined as Ctrough day2 (time point 3) and C_max_ day2 (time point 4), respectively. The fifth and sixth sets of blood sampling were performed on the day after the last cycle of chemotherapy to assess the long-term effects of chemotherapy and were defined as Ctrough day2* (time point 5) and C_max_ day2* (time point 6), respectively. These blood samples and blood coagulation tests were used to analyze the plasma concentrations of edoxaban, prothrombin time-international normalized ratio (PT-INR), activated partial thromboplastin time (APTT), and active factor Xa concentrations.

### Plasma concentration of edoxaban

Blood samples were collected by venous puncture and stored with heparin. Samples were immediately centrifuged at 2500 g for 10 min at 4 °C, and thereafter plasma was stored at -80 °C. Edoxaban concentrations were determined by Shin Nippon Biomedical Laboratories (Tokyo, Japan). Ultra-high performance liquid chromatography (HPLC) with tandem mass spectrometry (API 4000; AB Sciex Pte. Ltd., Tokyo, Japan) and an HPLC analytical column (CAPCELL PAK C18 MGII; 2.0 mm i.d. × 150 mm, 3 µm; OSAKA SODA Co., Ltd., Osaka, Japan) were used for the concentration analysis as previously reported [21]. The quantification range was 1–500 ng/ml.

### Blood coagulation tests

The PT-INR and APTT were analyzed as part of general blood coagulation tests. The PT-INR was obtained by multiplying the calculated prothrombin time (PT) with the international sensitivity index. PT was measured using Thromborel® S (Siemens, Munich, Germany) containing human placenta-derived thromboplastin and calcium, which initiate the coagulation reaction. APTT was measured using a Thrombocheck APTT-SLA (Sysmex, Kobe, Japan) containing synthetic phospholipids and calcium chloride. Both parameters were measured using an automated blood coagulation analyzer CS5100 (Sysmex, Kobe, Japan).

The concentration of active factor Xa was measured using a SensoLyte® Rh110 Factor Xa Assay Kit (#AS-72207; AnaSpec, Fremont, CA, USA), following the manufacturer’s instructions. The fluorescence intensity was measured using a microplate reader (SpectraMax iD3; Molecular Devices, San Jose, CA) to determine the concentration of Xa. The quantitative range was 0.0320–2.00 µmol/L.

### Endpoint measurements

The primary endpoint of this study was the percentage change in plasma edoxaban trough concentrations before and after chemotherapy initiation, which indicated the effect of chemotherapy on the excretion capacity of edoxaban.

The secondary endpoints included determining the percentage change in plasma edoxaban trough concentrations before chemotherapy initiations and after a series of chemotherapy cycles, which indicates the effect of multiple doses of chemotherapy on excretion capacity. The area under the plasma drug concentration–time curve (AUC) is a definite integral of the concentration of a drug in the blood plasma and reflects the actual exposure of the body to drugs after administration [22]. In this study, we calculated the AUC values of edoxaban before and two hours after administration using the linear trapezoidal method, which indicated the absorption capacity of edoxaban. The following formula was used to calculate AUC values:

$$\mathrm{AUC}=(\mathrm{Ctrough}+\mathrm{Cmax})\ast1/2:\ast2\mathrm h(\mathrm{ng}\ast\mathrm h/\mathrm{ml})$$

Percentage changes of AUC values, active factor Xa concentration, PT-INR, and APTT before and after chemotherapy were also calculated as secondary endpoints.

Any adverse events such as bleeding and thrombotic recurrence were recorded up to six months after initiating edoxaban administration. Bleeding events were defined as a combination of major or clinically relevant non-major bleeding events. Major bleeding events included a decrease in hemoglobin of 2 g/dL or more, requiring transfusion of two or more units of blood, occurring at a critical site, or contributing to death [23]. Clinically relevant non-major bleeding events did not meet the criteria for major bleeding but were associated with the need for medical intervention, such as contact with a physician or discontinuation of drug administration [24]. Recurrent thrombosis included cases identified by imaging studies with or without symptoms, and cases which resulted in death due to thrombus.

### Statistical analysis

The number of patients required for this study was calculated to be 14 to achieve a Type I error of 5% or less and a power of 80% or more in a one-tailed test. Therefore, considering cases of discontinuation during the study, the number of planned study participants was set at > 15. The lower limit (upper limit is ∞) of the 95% CI corresponding to a one-sided t-test of percentage change rate in edoxaban trough concentrations before and after chemotherapy administration is considered significant if it is greater than 0%. Each parameter, including the AUC values, PT-INR, APTT, and active factor Xa concentration between time points, was statistically analyzed using the Wilcoxon signed-rank test or Friedman test, and *P*-values were adjusted using the Bonferroni method for multiple comparisons. Continuous variables are expressed as mean ± standard deviation (SD). Statistical significance was set at *P* < 0.05. All statistical analyses were performed using R version 4.2.2 [25].

## Results

### Characteristics of participants

Patients with ovarian or uterine corpus cancer diagnosed with VTE and/or PE received anticoagulant therapy, including UFH or DOACs, preoperatively. After surgery, the patients were asked to participate in this study, and the 16 patients who provided written informed consent were included. The participants’ characteristics are listed in Table 1. The median age of the patients was 57 (interquartile range; 55–68) years, and the mean body mass index was 22.1 (interquartile range; 19.7–24.5). Among these, 12 patients had ovarian cancer and 4 had uterine corpus cancer. Of the 12 patients with ovarian cancer, 1 had serous carcinoma, 4 had endometrioid carcinoma, 6 had clear cell carcinoma, and 1 had mixed carcinoma (endometroid/serous). Of the four uterine corpus cancers, three were endometrioid carcinomas, and one was a carcinosarcoma. Eight patients were diagnosed at an early stage (stages I–II), while the remaining patients were diagnosed at advanced stages (stages III–IV). Twelve patients had VTE only and four had PE with VTE. Among those 4 cases, one case with ovarian cancer was found to have multiple cerebral infarctions and diagnosed as Trousseau's syndrome. Based on the body weight of the patients, three patients received 60 mg of edoxaban, and the remaining patients received 30 mg. None of the patients had a history of thrombosis or thrombotic predisposition. All patients strictly adhered to the edoxaban regimen prescribed during the study period.

**Table 1:** Characteristics of the trial population at baseline (*n* = 16)

Characteristics	Median (IQR)	
**Age (years)**	57 (55–68)	
	**Mean (IQR)**	
**BMI**	22.1 (19.7–24.5)	
	**No. (%)**	
**Type of cancer**		
Ovary	12 (75%)	
Uterus corpus	4 (25%)	
**Histology**	**Ovary**	**Uterus corpus**
Serous carcinoma	1 (6.3%)	0 (0%)
Endometrioid carcinoma	4 (25%)	3 (18.8%)
Clear cell carcinoma	6 (37.5%)	0 (0%)
Carcinosarcoma	0 (0%)	1 (6.3%)
Mixed carcinoma	1 (6.3%)	0 (0%)
**Stage of cancer**	**Ovary**	**Uterus corpus**
I	7 (43.8%)	1 (6.3%)
II	1 (6.3%)	0 (0%)
III	4 (25%)	2 (12.5%)
IV	0 (0%)	1 (6.3%)
**Location of VTE**		
DVT	12 (75%)	
PE + DVT	4 (25%)	
**Edoxaban administration**		
60 mg	3 (19%)	
30 mg	13 (81%)	
**History of previous thrombosis or thrombotic predisposition-no**	0	

*IQR* Interquartile range, *BMI* Body mass index, *VTE* Venous thromboembolism, *DVT* Deep vein thrombosis, *PE* Pulmonary embolism

### Percentage change of trough concentrations of edoxaban during chemotherapies with paclitaxel

**Fig. 2:**
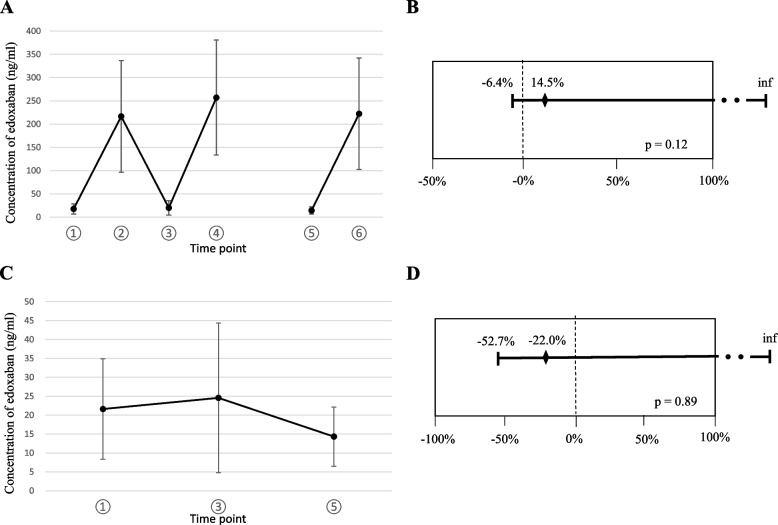
The percentage change in trough concentrations of edoxaban during chemotherapy with paclitaxel. **A** Concentrations of plasma edoxaban at each time point. Dots and error bars represent mean ± SD. **B** Percentage change in trough concentration of edoxaban from time points 1 to 3 (*n* = 15). Dots and error bars represent mean ± 95% CI. **C** Trough concentrations of plasma edoxaban at time points 1, 3, and 5 (*n* = 8). Dots and error bars represent mean ± SD. **D** Percentage change in trough concentrations of edoxaban from time points 1 to 5 (*n* = 8). Dots and error bars represent mean ± 95% CI. *CI*: confidence interval; *inf*: infinity

The primary endpoint of this study was to determine whether the trough concentration of edoxaban was affected by the administration of chemotherapeutic drugs, including paclitaxel. The mean and SD of the plasma concentrations of edoxaban at each time point are shown in Fig. 2A. Among the 16 patients enrolled, plasma samples were collected after chemotherapy initiation in 15 patients and after the last chemotherapy cycle in eight patients. The mean values of Ctrough day1 (time point 1), C_max_ day1 (time point 2), Ctrough day2 (time point 3), C_max_ day2 (time point 4), Ctrough day2* (time point 5), and C_max_ day2* (time point 6) were 17.7 ± 10.9, 216.6 ± 119.9, 20.0 ± 15.6, 257.1 ± 123.6, 14.3 ± 7.8, and 222.4 ± 119.6 ng/ml, respectively. In the 12 patients with ovarian cancer, the mean values of Ctrough day1 (time point 1) and Ctrough day2 (time point 3) were 14.9 ± 6.2 and 15.2 ± 7.4 ng/ml, respectively. In the four patients with uterine corpus cancer, the mean values of Ctrough day1 (time point 1) and Ctrough day2 (time point 3) were 26.8 ± 16.6 and 33.1 ± 25.2 ng/ml, respectively. The mean trough concentrations were not significantly different between different tumor types (Table 2), indicating that different chemotherapy regimens did not alter the trough concentrations. The overall trough concentration of edoxaban changed from 17.7 ± 10.9 ng/ml to 20.0 ± 15.6 ng/ml after the initiation of chemotherapy with paclitaxel (*n* = 15), and the mean percent change in edoxaban concentration was 14.5% (95% CI: -6.4–∞; *P* = 0.12) with an SD of 45.9% (Fig. 2B); this was contrary to expectations, and indicated that the trough concentrations of edoxaban, which represents excretion capacity, were not significantly increased by chemotherapy and paclitaxel administration. In eight patients whose blood samples were collected at time points 1, 3, and 5, the trough concentration of edoxaban changed from 21.6 ± 13.3 to 14.3 ± 7.8 ng/ml (Fig. 2C), and the mean percent change in edoxaban concentration was -22.0% (95% CI: -52.7–∞; *P* = 0.89) with a SD of 45.9% (Fig. 2D). This showed that the trough concentrations of edoxaban were not significantly affected by multiple cycles of chemotherapy.

**Table 2:** Concentration of plasma edoxaban (ng/ml) during the study

**Time point**	**All patients** (*n* = 16)	**Cancer type**		**Edoxaban dosage**	
		**Ovarian** (*n* = 12)	**Uterus corpus** (*n* = 4)	**60 mg** (*n* = 3)	**30 mg** (*n* = 13)
1	17.7 ± 10.9	14.9 ± 6.2	26.8 ± 16.6	15.6 ± 6.0	26.3 ± 22.4
2	216.6 ± 119.9	211.8 ± 93.2	229.9 ± 194.4	188.8 ± 78.2	330.4 ± 206.6
3	20.0 ± 15.6	15.2 ± 7.4	33.1 ± 25.2	16.0 ± 7.2	36.1 ± 30.4
4	257.1 ± 123.6	237.4 ± 82.5	311.3 ± 208.0	207.9 ± 68.8	453.7 ± 92.0
5	14.3 ± 7.8	12.8 ± 4.3	16.8 ± 12.7	11.9 ± 4.4	21.5 ± 13.9
6	222.4 ± 119.6	156.4 ± 53.4	332.3 ± 123.8	164.4 ± 51.6	396.5 ± 77.1

*All values are expressed as mean* ± *SD*

**Fig. 3:**
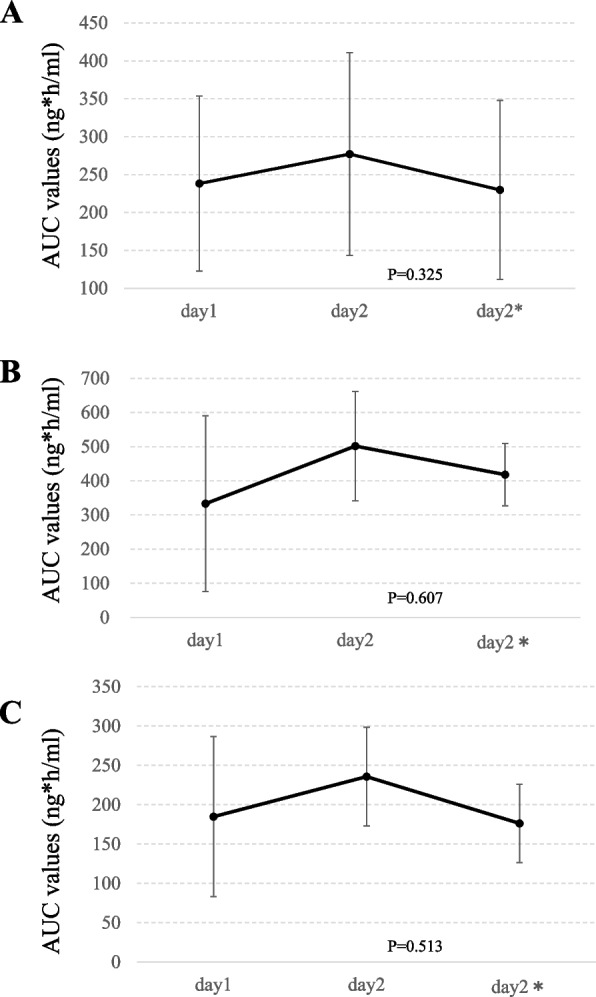
The AUC values of edoxaban administrated at day1 (before chemotherapy initiation), day2 (after chemotherapy initiation), and day2* (after the final chemotherapy session) (*n* = 8). **A** All cases. **B** Cases who received 60 mg of edoxaban (*n* = 2). **C** Cases who received 30 mg of edoxaban (*n* = 6)*.* Dots and error bars represent mean ± SD

AUC values that reflected the actual body exposure to edoxaban two hours after administration were calculated on day1 (before chemotherapy initiation), day2 (after chemotherapy initiation), and day2*(after the final chemotherapy session) from eight patients whose blood samples were collected at all time points. The average AUC values on day1, day2, and day2* were 238.2 ± 115.6, 277.1 ± 133.5, and 229.8 ± 118.2 ng*h/ml, respectively. These AUC values were not significantly affected by multiple cycles of chemotherapy (*P* = 0.325) (Fig. 3A). Among the two patients who received 60 mg of edoxaban, the average AUC values on day1, day2, and day2* were 330.0 ± 257.3, 501.7 ± 160.2, and 418.0 ± 90.9 ng*h/ml, respectively (*P* = 0.607) (Fig. 3B). Among six patients who received 30 mg of edoxaban, the average AUC values on day1, day2, and day2* were 184.8 ± 101.6, 235.7 ± 62.9, and 176.2 ± 49.9 ng*h/ml, respectively (*P* = 0.513) (Fig. 3C). The AUC values revealed that actual body exposure to edoxaban was not altered by chemotherapy at either of the edoxaban dosages.

### Percentage changes of active factor Xa concentration, PT-INR, and APTT during chemotherapy with paclitaxel

**Fig. 4:**
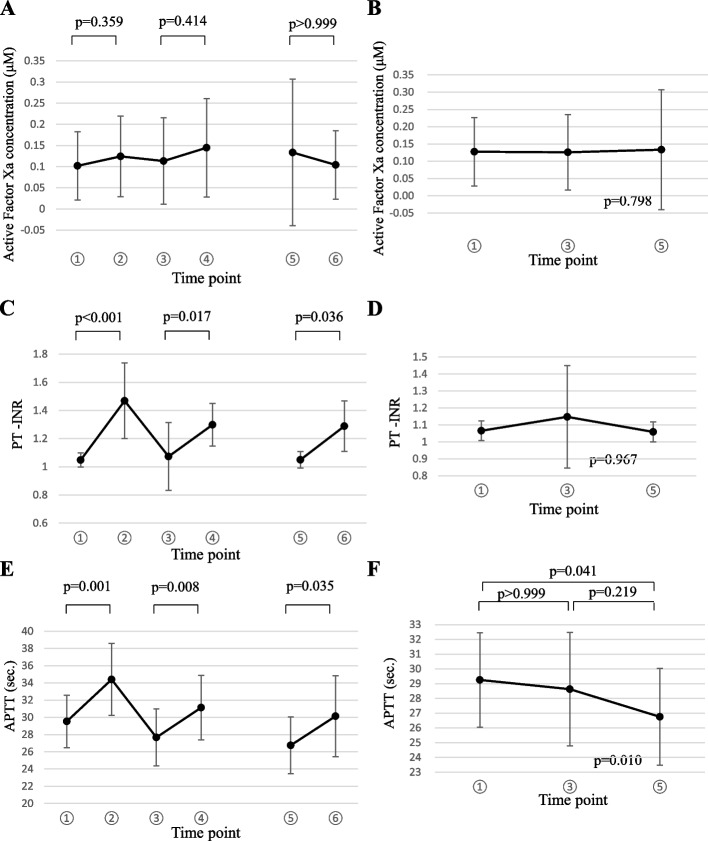
Percentage changes in active factor Xa concentration, PT-INR, and APTT values. **A** Active factor Xa concentrations at all time points. **B** Active factor Xa concentrations at time points 1, 3, and 5 (*n* = 8). **C** PT-INR values at all time points. **D** PT-INR values at time points 1, 3, and 5 (*n* = 8). **E** APTT values at all time points.** F** APTT values at time points 1, 3, and 5 (*n* = 8). Dots and error bars represent mean ± SD

The plasma concentrations of active factor Xa during the study are shown in Fig. 4A. The active factor Xa concentrations before (time point 1) and two hours after edoxaban administration (time point 2) were 0.102 ± 0.080 and 0.124 ± 0.095 μM (*n* = 16), respectively. One day after chemotherapy initiation, the active factor Xa concentrations before (time point 3) and two hours after edoxaban administration (time point 4) were 0.113 ± 0.102 and 0.145 ± 0.116 μM (*n* = 15), respectively. One day after the final chemotherapy session, the active factor Xa concentrations before (time point 5) and two hours after edoxaban administration (time point 6) were 0.133 ± 0.173 and 0.104 ± 0.08 μM (*n* = 8), respectively. In eight patients whose blood samples were collected at all time points, these values were not significantly altered during the study period (*P* = 0.359) (Fig. 4B).

The PT-INR values during the study period are shown in Fig. 4C. The PT-INR values before (time point 1) and two hours after edoxaban administration (time point 2) were 1.05 ± 0.05 and 1.47 ± 0.27 (*n* = 16), respectively. One day after chemotherapy initiation, the PT-INR values before (time point 3) and two hours after edoxaban administration (time point 4) were 1.07 ± 0.24 and 1.30 ± 0.15 (*n* = 15), respectively. One day after the final chemotherapy session, the PT-INR values before (time point 5) and two hours after edoxaban administration (time point 6) were 1.05 ± 0.06 and 1.29 ± 0.18 (*n* = 8), respectively. Overall, the PT-INR values increased two hours after edoxaban administration (*P* = 0.036) and returned to a similar level the following day. In eight patients whose blood samples were collected at all time points, the PT-INR values were not affected by chemotherapy (Fig. 4D).

The APTT values obtained during the study are shown in Fig. 4E. The APTT values before (time point 1) and two hours after edoxaban administration (time point 2) were 29.5 ± 3.0 and 34.4 ± 4.2 s (*n* = 16), respectively.. One day after chemotherapy initiation, the APTT values before (time point 3) and two hours after edoxaban administration (time point 4) were 27.7 ± 3.3 and 31.1 ± 3.7 s (*n* = 15), respectively. One day after the final chemotherapy session, the APTT values before (time point 5) and two hours after edoxaban administration (time point 6) were 26.8 ± 3.3 and 30.1 ± 4.7 s (*n* = 8), respectively. Overall, the APTT values were slightly extended two hours after edoxaban administration (*P* = 0.035) and returned to a similar level the following day. The APTT values for the various time points were slightly shortened compared with that of time points 1 and 5 (*P* = 0.041); however, there were no other differences among the time points (Fig. 4F).

### Safety profiles

No adverse events, including bleeding or recurrent thrombosis, occurred within six months of study. Outside of the set observation period, there was one case of hemorrhage and one case of recurrent thrombosis. One patient presented with intermittent epistaxis that quickly resolved by applying pressure; therefore, oral edoxaban was not discontinued. In one patient with a recurrent thrombus, a thrombus in the lower inferior vena cava was observed nine months after the administration of edoxaban, and anticoagulation therapy was changed from edoxaban to heparin.

## Discussion

The treatment of VTE in patients with active cancer remains challenging owing to complications such as an increased risk of bleeding and potential drug-drug interactions with chemotherapy. Although DOACs have significantly fewer drug-drug interactions than VKAs, drugs that strongly affect the CYP3A4 enzyme and/or P-glycoprotein can alter the plasma concentration of DOACs and lead to clinically significant alterations in their anticoagulant effects [17]. The safety and efficacy data of edoxaban is comparable to that of dalteparin in the Hokusai-VTE Cancer study in patients with non-gastrointestinal cancers; this suggests that the drug-drug interactions between DOACs and anticancer agents are clinically manageable [26]. However, concrete safety data regarding the use of DOACs in cancer patients receiving chemotherapies indispensable for the cure are still lacking. This study is the first to monitor the plasma concentration of edoxaban in patients with ovarian and uterine corpus cancer who were receiving chemotherapy with paclitaxel, and to evaluate the percent change in trough concentrations and AUC values of edoxaban before and after treatment. The trough concentrations and AUC values of edoxaban were shown not to be significantly altered by multiple cycles of chemotherapy, indicating that edoxaban is an acceptable option for the treatment of CAT in patients with gynecological cancer receiving chemotherapy, including paclitaxel.

Many chemotherapeutic drugs induce or inhibit the activity of CYP3A4, P-glycoprotein, or both. These include anthracyclines such as doxorubicin; antimycotic agents such as vincristine and paclitaxel; topoisomerase inhibitors such as topotecan and etoposide; alkylating agents such as cyclophosphamide; tyrosine kinase inhibitors such as imatinib, lenvatinib, and sunitinib; immune-modulating agents such as cyclosporine and tacrolimus; and hormonal agents such as tamoxifen and anastrozole [27]. In contrast, platinum-based agents, including carboplatin; intercalating agents; and monoclonal antibodies, including bevacizumab, have not been reported to have significant inhibitory or inducing effects on CYP3A4 or P-glycoproteins [17]. The paclitaxel and carboplatin regime, with or without bevacizumab, is the most frequently used regimen for the treatment of ovarian and uterine corpus cancers; therefore, the use of paclitaxel might affect the plasma concentration of DOACs, which may interfere its treatment effect. Previously studies on the efficacy of DOACs in treating CAT have not focused on gynecological malignancies; this is most likely due to the few cases included in these studies. In the Hokusai-VTE Cancer study, only 19 and 15 cases of ovarian and uterine corpus cancers, respectively, were included in the edoxaban group, whereas 33 and 22 cases were included in the dalteparin group [11]. In that study, only 40 cases in the edoxaban group and 47 cases in the dalteparin group received taxanes during the observation period [11]. Therefore, the safety and efficacy of edoxaban in patients with gynecological cancer receiving chemotherapy requires investigation. Furthermore, although several phase III studies have shown the comparable safety and efficacy of DOACs in patients with cancer, none have validated the plasma concentration of each DOAC during chemotherapy. Herein, we measured the specific changes in plasma edoxaban concentrations before and after chemotherapy.

In this study, percentage changes in the trough concentrations of edoxaban before and after chemotherapy were set as the primary endpoints as the bleeding risk in patients receiving edoxaban orally was reported to correlate more strongly with its plasma trough concentration than with AUC or C_max_ values [28]. Although the number of participants was small due to the high cost of measuring the edoxaban concentrations, we successfully showed that the percentage change in trough concentration was not significantly increased the day following chemotherapy (14.5%; 95% CI: -6.4–∞; *P* = 0.12).

The AUC values, PT-INR, APTT, and plasma concentration of active factor Xa were evaluated as secondary endpoints. The PT-INR and APTT values were altered two hours after edoxaban administration, as previously reported [29], and returned to similar levels one day after administration. Throughout time points 1, 3, and 5, no significant changes in AUC values, PT-INR values, or plasma concentration of active factor Xa were observed. Although APTT values were slightly shortened from time points 1 to 5 (*P* = 0.041), these results suggest that concomitant chemotherapy with paclitaxel and edoxaban did not induce hypercoagulability in patients.

This study has several limitations. First, this was a single-institution study with a small sample size. Second, no reference values are available for percentage change in plasma edoxaban concentration exist owing to the lack previous studies on this topic. Third, it was necessary to evaluate the percentage change in plasma concentration from only six blood-sampling time points as this was invasive for participants and the cost of measuring plasma edoxaban and active factor Xa concentration levels were high. A greater number of patients with more blood sampling is desirable to confirm the safety of edoxaban during chemotherapy.

## Conclusion

Patients with CAT and ovarian or uterine corpus cancer who were receiving edoxaban orally showed no significant increase in the trough concentration of edoxaban while undergoing chemotherapy, including paclitaxel, for cancer treatment. These findings suggest that edoxaban is safe for the treatment of CAT during the treatment of gynecological cancers using chemotherapy.

## Data Availability

The datasets used and/or analyzed in the study are available upon reasonable request from the corresponding author.

## Abbreviations

CI: Confidence interval
CAT: Cancer-associated thrombosis
LMWH: Low-molecular-weight heparin
UFH: Unfractionated heparin
VKA: Vitamin K antagonist
DOAC: Direct oral anticoagulant
VTE: Venous thromboembolism
PE: Pulmonary embolism
AUC: Area under the curve
APTT: Activated partial thromboplastin time
PT-INR: Prothrombin time-international normalized ratio
SD: Standard deviation
